# Emotion and International Business: Theorising Fear of Failure in the Internationalisation

**DOI:** 10.3389/fpsyg.2022.850816

**Published:** 2022-03-22

**Authors:** Rebecca Kechen Dong

**Affiliations:** UniSA Business, University of South Australia, Adelaide, SA, Australia

**Keywords:** fear of failure, decision-making, internationalisation process of a firm, emotion, international business (IB)

## Abstract

The road to internationalisation is paved with risk, uncertainty, the possibility of failure, and the Coronavirus Disease-19 (COVID-19) phenomenon. However, the process of internationalisation (IP) theory treats an individual decision-maker as a “black box.” Emotions are largely ignored by international business (IB) researchers. This study offers conceptual thoughts on the role of fear of failure in the process of internationalisation. It argues that managers experience this emotion in making internationalisation decisions for a firm, which is an area of study that requires further understanding. Following the content analysis method in the literature review and a theory-based adaptation approach to complete the conceptualisation, this manuscript joins the scholarly conversations on “connecting the IP model to people” and “emotion and internationalisation.” Focussing on fear of failure as a new perspective, this manuscript contributes to IB literature by suggesting new avenues in understanding decision-making about international activities by embracing psychological insights. It also contributes to IB practitioners by offering implications for understanding one’s emotional state and its effect on decision-making about internationalising ventures.

## Introduction

Globalisation of the world economy has created international opportunities. Nevertheless, taking the current international business (IB) environment into consideration, the global infection status of Coronavirus Disease-19 (COVID-19) had brought risks on economic activities (Yue et al., 2020) and mental health disorders to individuals (Chen et al., 2021; Pappa et al., 2021; Zhang et al., 2022). Internationalisation is instrumental to a firm’s survival and growth along with the potential for a positive effect on economic development (The World Bank, 2019). While internationalisation may contribute to the improved performance of a firm (Lu and Beamish, 2006), a firm’s internationalisation activities comprise both progress and setbacks (Clarke and Liesch, 2017; Kriz and Welch, 2018), in particular, facing frequently occurring challenges or failure in international markets (Alexander and Korine, 2008), de-internationalisation (Tang et al., 2021), and host country risk (Tang and Buckley, 2020). Failure in international business refers to unexpected decreased involvement in international activities, divestment, and export withdrawal (Nummela et al., 2016) and is considered a natural part of the process of the internationalisation of a firm as it comprises both progress and setbacks (Clarke and Liesch, 2017; Kriz and Welch, 2018).

To support the sustained internationalisation efforts of firms, it is important to consider the role of decision-makers is critical to the firm’s internationalisation activities because their decisions determine the subsequent choice of foreign markets and the degree of internationalisation (Lu and Beamish, 2001). Individual-level factors relevant to the business owner/decision-maker in internationalisation are argued to be important, such as personal attributes and skills (Manolova et al., 2002), risk perception (Figueira-de-Lemos et al., 2011; Clarke and Liesch, 2017), the perception of the environment (Cavusgil, 1984), and tolerance of ambiguity (Acedo and Jones, 2007). Psychology literature emphasises the importance of emotion regarding its impact on decision-making (Damasio, 1994; Clore and Huntsinger, 2007, 2009). However, the topic of emotion remains under-researched in the IB literature (Meyer and Gelbuda, 2006; Van de Laar and De Neubourg, 2006). The emerging empirical studies reveal that fear of failure, a particular emotion, has impacts on the exporting behaviour of SMEs (Alon et al., 2013) and de-internationalisation decisions (Lafuente et al., 2015). While the exploration of fear of failure in the IB literature is still scant, this topic receives great interest from entrepreneurship scholars. For instance, fear of failure is one of the antecedents to decision-making (Shepherd et al., 2015) with subsequent effect on influencing entrepreneurs’ actions throughout the entrepreneurship process (Ucbasaran et al., 2009; Cacciotti et al., 2016).

The process of internationalisation (IP) model (also called *the Uppsala model*) (Johanson and Vahlne, 1977, 2009; Vahlne and Johanson, 2017) highlights the required entrepreneurial nature and draws multiple parallels between entering international markets and a new venture start-up. However, as opposed to entrepreneurship research, which is grounded at the individual level, the IP model treats the role of an individual decision-maker as a “black box” (Vahlne and Johanson, 2017, p. 1,089). This study is aimed at joining a conversation about “connecting the internationalisation process to people” to enhance our understanding of the individual decision-maker at the micro-level of the internationalisation process of a firm (Coviello et al., 2017). Vahlne (2020) calls for future research to apply psychological findings on their research issues regarding the IP model. To answer this research call, this manuscript proposes fear of failure, an individual-level factor, that is likely to influence firm-level outcomes. This manuscript argues the need to explore the individual decision-maker’s emotions when experiencing fear of failure in the context of the internationalisation of a firm. Underlying the existing studies, which illustrate the link, is clear between fear of failure and noticing business opportunities (Anokhin and Abarca, 2011; Li, 2011), motivation (McClelland and Watson, 1973; Morgan and Sisak, 2016), risk perception (Arenius and Minniti, 2005), and taking action to exploit opportunities (Choi and Shepherd, 2004; Westhead, 2008; Hmieleski and Baron, 2009).

The core aim of this manuscript was to recognise fear of failure as an important factor for the individual decision-makers in the IP model (Vahlne and Johanson, 2017). This manuscript is motivated by a number of limitations of the existing IB literature and the needs of business managers who underwent the internationalisation process. Furthermore, this manuscript is motivated by addressing managerial needs. Fear of failure could be an unconscious experience when individuals are not aware of what they fear (Loewenstein et al., 2001). This study contributes to facilitating decision-makers to be aware of the fear of failure. To achieve the above aims, this manuscript has applied content analysis for conducting IB literature review (Gaur and Kumar, 2018) management studies (Duriau et al., 2007). As such, this manuscript is structured into two lines of argument to generate a theoretical conceptualisation. The first line includes a literature review on internationalisation, following a discussion on its micro-foundations. This part identifies individual-level factors that drive the internationalisation of a firm. The second line of argument discusses the fear of failure drawn from psychological and entrepreneurship literature, offering a deepened understanding of why this particular emotion matters within the context of making internationalisation decisions. By revising extant knowledge, this study follows a theory-based adaptation approach to complete the conceptualisation (Jaakkola, 2020). Thereafter, it concludes with a summary of the contributions of the study and offers directions for future research.

## Review the Uppsala Model of Internationalisation

Internationalisation is regarded as the process of a firm’s gradually increasing engagement in foreign markets (Johanson and Vahlne, 1977, 2009; Welch and Luostarinen, 1988; Vahlne and Ivarsson, 2014). The IP model provides fundamental insights into the internationalisation behaviour of the firm. The IP model is widely cited in the IB scholarship. The authors of the IP model have revised their work frequently to cater to the changing globalisation phenomenon and the advancement in the IB literature.

First, this manuscript summarises the evolution of the IP models in Table 1. It locates and contextualises this theory by summarising how and why it develops and updates the earlier versions. This study offers a comparison of their representative models in the following discussion: the original IP model (Johanson and Vahlne, 1977); the current widely recognised IP model (Johanson and Vahlne, 2009), and the newest IP model (Vahlne and Johanson, 2017). It then focuses the discussion on the latest version of the IP model and on how other scholars contribute to extending the IP model.

**TABLE 1:** Evolution of the process of internationalisation (IP) models.

Uppsala literature	Focus	Factors in state aspect	Factors in change aspect	Key findings to explain the state and change mechanism
Johanson and Vahlne (1977) (original model)	Learning	Market knowledge; market commitment	Commitment decisions. Current activities	Internationalisation is viewed as a dynamic learning process. Firms learn international market knowledge about entering a foreign market. Incremental decisions enhance the scope of internationalisation.
Johanson and Vahlne (2003)	Network	Market knowledge; market commitment	Commitment decisions. Current activities	Add network relationship development in internationalisation. Firms learn knowledge in their relationships. Learning contributes to increasing firms’ commitment to international markets.
Johanson and Vahlne (2009) (widely-recognised IP model)	Network	Knowledge opportunities network position	Relationship commitment decisions: learning, creating, and trust-building	International business environment is viewed as a “web of relationships.” Firms develop new knowledge from relationships. With a firm’s increasing involvement in internationalisation, it will build more overseas networks and learn more about the international environment. With the accumulation of experiential knowledge, market commitment and trust, it allows a firm to overcome the uncertainty, risk and a lack of opportunity awareness that constrains their internationalisation activities
Vahlne et al. (2012)	Network	Knowledge, opportunities, and entrepreneurial capabilities. network (external and internal)	Relationship commitment decisions: Learning, creating, and trust-building	The internationalisation is conceptualised for the global firm and hierarchically acting headquarters (HQ). Uncertainty is highlighted in the HQ management in the global firm, due to a liability of outsidership. Networks include external and internal partners (subsidiaries). Two parties can influence each other’s decision. Learning is not sufficient because HQs have difficulty in informing the subsidiary directly.
Vahlne and Johanson (2013)	Network	Dynamic capabilities, operational capabilities. network position	Commitment decisions (reconfiguration and change of coordination); inter-organisational processes (learning, creating, and trust-building)	The internationalisation is underpinned for emerging new phenomenon: the multinational enterprise (MNE) and foreign direct investment (FDI). Firms can improve organisation capability by enhancing capabilities including opportunity development, internationalisation, and networking. Firms can strengthen the network position by improving the inter-organisational and the intra-organisational network position, and network power. Commitment decisions can be expressed by volume and degree of restraint in re-allocating the resources. They are regarded as reconfiguration and change of coordination.
Vahlne and Ivarsson (2014)	Globalisation	Capabilities; commitment/performance	Commitment decisions; organisational processes (learning, creating, and trust-building)	The IP model is underpinned for the globalisation phenomenon by Multinational Enterprise (MNE). Add technology development capability and globalisation-capability can improve the organisation capability. Firms can improve performance with the degree of globalisation, including geographical configuration and coordination.
Vahlne and Johanson (2017)	Globalisation	Capabilities; commitment/performance	Commitment processes; knowledge development processes	Internationalisation is updated by considering modern firms, multinational business enterprise (MBE). A decision to reconfigure resources influences the firm’s capabilities, resource and its network partner, and subsequently performance in international markets. A changed capability and resource will further influence knowledge development process, decision-making and resource allocation.

**FIGURE 1:**
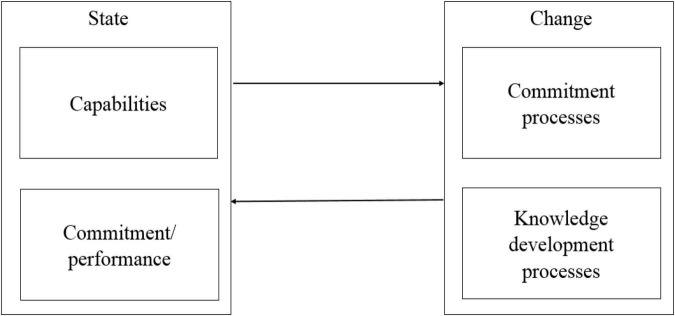
The process of internationalisation (IP) model (Johanson and Vahlne, 2017).

All of the IP models in different literature (Table 1) share the same mechanism underlying the theory: the relationship between “state and change” (Johanson and Vahlne, 1977, 2009; Vahlne and Johanson, 2017). They explain that internationalisation is a constantly ongoing process where the “state” aspect and the “change” aspect interact with each other (Figure 1). The “change” aspect of the IP model concerns the firm’s behaviours in the form of the firm’s degree of commitment. The variables from the “change” aspect are essential elements where the action takes place.

Vahlne and Johanson (2017) developed the newest IP model (Figure 1) as motivated by the globalisation phenomena underpinning risk, uncertainty, and partial ignorance by today’s managers. There are two starting points for the change aspect: one is the decision processes related to committing; the other one is the continuous knowledge development processes. The commitment decisions are defined as the product of the size of the investment and its degree of inflexibility (Johanson and Vahlne, 2009, p. 1,412). Managers make the decisions related to the firm’s commitment to internationalisation, and they need to draw on experience and knowledge of opportunities to proceed with the commitment decision. It should be noted that there are sub-processes under the knowledge development processes and commitment processes from the 2017 IP model (Vahlne and Johanson, 2017). For example, learning, creating, and trust-building are changing variables, representing sub-processes inside the knowledge development process. These factors are occurring continuously and affecting each other. In turn, they will change the state aspect of the IP model that includes the firm’s capabilities and resource commitment. In the meantime, the changed resource commitment may be reflected as a reduced commitment, comparable to leaving a market, reducing diversification, or discontinuing a relationship. As a result, a changed capability and performance will make differences to further knowledge development, decision-making, or resource allocation.

The newest IP model is an advancement on the original model (1977) and the later well-recognised model (2009). The original IP model focuses on learning the psychic distance constrains firm’s learning and the incremental commitment to internationalisation. Psychic distance means “the sum of factors preventing the flow of information to and from the market” (Johanson and Vahlne, 1977, p. 24). The commitment decisions are based on different knowledge, and knowledge is conclusive to a firm’s growth (Penrose, 1995). Unfortunately, human beings suffer from constraints on information processing capacity (Cyert and March, 1963). Furthermore, the most cited IP model (Johanson and Vahlne, 2009) focuses on relationships. Internationalisation often occurs when the firm may still lack the knowledge, information, experience of foreign institutions and markets, and reliable networks. The internationalisation of a firm starts with international opportunity – “the chance to conduct an exchange with new partners in new foreign markets” (Ellis, 2011). Firms need networks to access information and knowledge of risk and uncertainty concerning the opportunities in foreign markets (Ellis, 2011). However, firms suffer the liabilities of foreignness and of outsider status, especially, at the early stages of the internationalisation process. The liability of foreignness (Denk et al., 2012) leads to the additional tacit and social costs that foreign firms face when entering a particular host market, costs not incurred by well-embedded indigenous companies (Zaheer, 2002). The liability of outsider status is an outcome of the absence of a relevant network position inside the market (Johanson and Vahlne, 2009). As such, the lack of a network is identical in constraining a firm’s commitment to internationalisation.

Although each version of the IP model has a different focus: learning (Johanson and Vahlne, 1977), relationship (Johanson and Vahlne, 2009), and globalisation (Vahlne and Johanson, 2017), the common points relate to shedding light on risk and uncertainty of the operational context for the firm’s internationalisation (Liesch et al., 2011). Liesch et al. (2011) suggest that risk refers to the possible action outcome, especially, the possible losses, while uncertainty focuses on an individual’s confidence in meeting their estimates or expectations. Risk, in the context of the internationalisation process, is defined as “the extent to which firms may lack knowledge about whether potentially significant and or disappointing outcomes of its decisions will be realised” (Clarke and Liesch, 2017, p. 927). This study supports the above understanding of risk and uncertainty, which highlights their relevance to decision-making. In particular, it implies that risk in internationalisation relates to the decision-maker’s expectation and emotional attachment, which are reflected in their description of “disappointing outcomes of decisions.” However, these definitions are complicated. Due to the fact that the boundaries of risk and uncertainty are wide-ranging, such definitions of risk and uncertainty are not unified in different research fields, such as Entrepreneurship and Psychology. Therefore, this manuscript relies on the Oxford English Dictionary’s definitions of the concept of risk: “a situation involving exposure to danger” (Oxford Dictionaries, 2019a); and the concept of uncertainty: “the state of being uncertain, can be referring to something that is uncertain or that causes one to feel uncertain” (Oxford Dictionaries, 2019b). By comparing with other applications, the definitions of risk and uncertainty in the Oxford dictionary are more explicit and easier to comprehend.

## Making Extensions to the Uppsala Model

There are four important levels of analysis that are required to empirically research internationalisation phenomena: the role of the individual manager, the firm level, the industry level, and the environment level (Buckley and Lessard, 2005). In most IB studies, research focussing on firm-level studies makes it difficult to understand the role of individuals in the context of making internationalisation decisions. The IP model (2017) considered the role of the individual as a core micro-foundation of the internationalisation process. It clarified the level of research as based on the IP model operating at the micro-level, the level of each firm. The changes at the micro-level (firm-level activities) result from the mille- to micro-level (the level of individuals or subgroups within the organisation). Indeed, their work includes the mille- to micro-level assumptions but does not reflect the role of individuals in their descriptions of a firms’ internationalisation process (Vahlne and Johanson, 2017, p. 1,089). By doing so, the concept of evolution is the sum of changes happening to people (the mille- to micro-level) but aggregated to the level of the firm. In summary, the key point here is that it is the individual’s change in their actions (at the mille- to micro-level) that is driving the firm’s internationalisation activity (at the micro-level).

The IP model’s inability to explain the role of individuals has been noted in emerging IB literature. The internationalisation phenomenon is conceptualised as a process of the entrepreneur’s behaviours taken in time (Jones and Coviello, 2005). To understand internationalisation, there is a need to understand individual-level factors pertinent to the decision-maker/owner/founder in the internationalisation of a firm (Manolova et al., 2002). A significant suggestion in researching the new internationalisation theory is to attend fully to the individual-level influence and their impact on firm-level outcomes (Coviello et al., 2017). Certain IB scholars provide insight into extending the IP model, building up a conversation on “connecting people to internationalization” (Table 2).

**TABLE 2:** Extensions to the process of internationalisation (IP) model.

Literature	Added factors	Key findings contributing to explain the internationalisation process
Clarke and Liesch (2017) extend IP Model (2009)	Added risk perception and risk propensity.	• Firms pursue a wait-and-see strategy to address risk management. • The individual decision-maker’s risk behaviour on the risk levels of the firm. • The decision-maker needs to re-evaluate the firm’s strategic options according tolerance of existing risks in commitment decisions.
Coviello et al. (2017) extend IP Model (2017)	Added micro-level influence (decision-makers) and macro-level	• Change processes from IP model (2017) occur at the level of an individual decision-maker.
	Influence (digitisation).	• Individual’s decisions and behaviours are shaped by bounded rationality and bounded reliability. • Decision-makers’ rationality and reliability determine both change and state variables from IP model to some extent. • Firm must possess efficient governance mechanism in the internationalisation activities.
Dow et al. (2018) extend IP Model (1977, 2009, and 2014)	The inertial state variables and enabling state variables.	• Managers engage with initial “change” aspect to drive internationalisation activities. They make decisions to commit resources to international markets. • Inertia limits the self-reinforcing cycle of the internationalisation: the passage of time and the accumulation of experiential knowledge may not lead to positive internationalisation outcome. • Inertia includes three aspect: specialised assets, network, and individual-level biases.

Table 2 summarises the new extensions to the IP model (Clarke and Liesch, 2017; Coviello et al., 2017; Dow et al., 2018). They all highlight the role of individuals in the IP and make contributions to addressing the limitation of IP models (Johanson and Vahlne, 1977, 2009; Vahlne and Johanson, 2017). It is essential to understand the difference of decision-makers’ behaviours and their unique situations in the internationalisation process (Coviello et al., 2017). This study integrates their implications of “connecting the internationalisation to people” as follows:

First, it argues a need to look at individual-level factors in the context of making the internationalisation decisions of a firm. The micro-foundation refers to “locating the causes of a phenomenon at a level of analysis lower than the phenomenon itself, to look at how behaviour of individual members within teams/units/firms at the micro-level of organisation, influence organisational constructs at the macro-level” (Coviello et al., 2017, p. 1,155). Their argument is that the characteristics and actions of individuals are tightly connected to the firm’s internationalisation outcomes. Second, it is important to address the role of individual decision-maker in internationalisation that includes the decision-maker’s personality, cognitive adaptability, and social competencies (Coviello et al., 2017), the manager’s international actions (Dow et al., 2018), and the decision-maker’s perception of risk and uncertainty as inherent to a new market entry (Knight and Liesch, 2016, p. 98). The third implication is to apply other disciplines’ knowledge to study the internationalisation process. When exploring the theoretical issues in early internationalisation, it should be noted that IB is a derivative field that requires drawing on a wide range of other fields and disciplines (Knight and Liesch, 2016, p. 98). Firms expand incrementally abroad in terms of commitment (investment) and the choice of destination countries (Johanson and Vahlne, 1977, 2009; Vahlne and Johanson, 2017). That journey is supported by certain behavioural processes of individuals (Chandra et al., 2009; Chandra et al., 2012). By comparison, the IP and the process of entrepreneurial action (Wood et al., 2012), share the same logic, both starting with making the decision to pursue the opportunity of entering a new market (Chandra et al., 2012)/international market (Johanson and Vahlne, 1977, 2009; Vahlne and Johanson, 2017), namely, pursuing international opportunities across national borders (Reuber et al., 2018). This suggests that “we need a great understanding of the individuals that are central to the firm’s internationalisation behaviour” (Oviatt and McDougall, 2005, p. 17), so drawing on concepts and theory from the entrepreneurship and psychology literature.

The above implications signal that it is essential to “connect the internationalisation to people” by locating the role of individual decision-maker in the centre of a firm’s internationalisation activities, which matters in the context of making the internationalisation decisions of a firm (Coviello, 2015; Coviello et al., 2017). It is an individual’s internationalisation decision that is relevant to the assessment of an international opportunity (Chandra, 2017) and making the choice between foreign markets (Clark et al., 2018) and the degree of internationalisation preferred by small firms (Lu and Beamish, 2001). Decision-making means the “entire process of choosing a course of action” (Hastie, 2001, p. 657). This study proposes that the individual decision-maker’s choice of action to follow will influence the commitment processes from the change aspect of the IP model (2017). However, a majority of IB studies are only focussing on the cognitive aspect inherent within the decision-making. The topic of emotion has been silent in internationalisation studies since 2006 (Meyer and Gelbuda, 2006; Van de Laar and De Neubourg, 2006), even though emotion is an important part of everyday experience and has a prominent role in the psychology field. Other scholars offer individual-level psychology-informed insights to inform the interaction between emotion and cognition and its influence on making decisions. Central to the business that is mostly personal experience based are the individual’s feelings and emotions (Morris et al., 2012).

**FIGURE 2:**
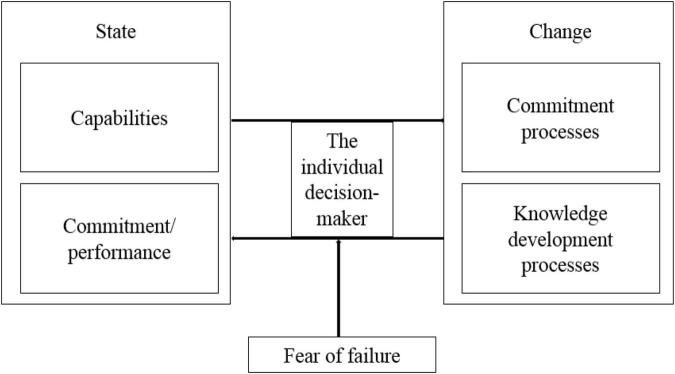
The conceptual model: building the link between fear of failure and the process of internationalisation.

Given the fact that emotion and cognition are interrelated in the decision-making to determine one’s choice of action taking (Lerner and Keltner, 2000), the environment is interpreted both emotionally and cognitively (Clore and Huntsinger, 2007, 2009). In this study, the author proposes to include a specific emotion factor, fear of failure, in theorising the IP model (2017) due to its relevance to the individual decision-maker in the context of making internationalisation occur (Figure 2). The reasons are justified in the following sections.

## Emotion and the Context of Decision-Making

Emotion and cognition are associated with different parts of the brain, consequently contributing to influencing one’s behaviours. Cognition means “all processes by which sensory input is transformed, reduced, elaborated, stored, recovered, and used” (Neisser, 1976, p. 4). It means an individual’s belief about the linkage between the choice of actions and the subsequent outcomes of those actions (Gavetti and Levinthal, 2000). Social cognition is defined as “the perception of others, the perception of self, and interpersonal knowledge” (Beer and Ochsner, 2006, p. 99). The notion of social cognition implies that a decision-maker’s thinking is linked to the external objects rather than only focussing on the “self.” The *affect-as-information theory* (Clore and Huntsinger, 2007) and the *affective processing principle* (Clore and Huntsinger, 2009) explain that people interpret the environment both emotionally and cognitively. Here, affect is a broad term referring to emotions and moods in general, such as happiness, sadness, fear, and anger. Clore and Huntsinger (2007, p. 393) state that “affective cures of mood and emotion influence judgements directly by serving as experiential and bodily information regarding how one feels about the object of judgement.” The fundamental assumption behind the theory is that the emotion being experienced is a reaction to the object to be judged. Individuals make decisions in a particular environment, starting from cognitive assessments on a potential outcome based on their embedded environment. They perceive the potential outcome as an environment cue, such as perceiving an environmental factor as a threat. It then triggers an individual’s emotions. In turn, emotion works back to the cognitive assessment to impacts how an individual interprets the information. When individuals are experiencing emotions, they will attach their personal value to assess the object (Schwarz, 1990). Emotion may play a more critical role in complex environmental settings when compared to cognition (Forgas, 1995). When cognitive assessments and emotions contradict each other, an individual’s behavioural choice is often driven by emotion (Loewenstein et al., 2001). Consequently, cognition and emotion interplay to frame decisions, determining an individual’s behavioural choices. The author summarises the empirical findings by reviewing the literature concerning emotions and decision-making in Table 3.

**TABLE 3:** Literature review of emotion in the context of decision-making.

Research field	Main research outcome	Illustrative empirical studies
Psychology	• Emotions are incorporated into an individual’s decision-making process, affecting an individual’s cognitive and behavioural responses. • The environment cues trigger an individual’s emotion. • Emotions shape one’s judgements or choices involving risk.	Flam, 1990; Damasio, 1994; Lerner and Keltner, 2000; Lerner and Keltner, 2001; Loewenstein et al., 2001; Smith et al., 2002
Entrepreneurship	• The process of entrepreneurship integrates emotional experiences. • The entrepreneurial environment can generate emotional cues, affecting their judgement on an opportunity of entering a new market. • Entrepreneurs balance the linear and non-linear (emotion) thinking style to process information.	Grichnik et al., 2010; Foo, 2011; Groves et al., 2011; Li, 2011; Shepherd et al., 2011; Morris et al., 2012; Welpe et al., 2012;
International business	• The process of internationalisation incorporates emotions. • Emotional responses affect making internationalisation decisions in their organisation. Emotion and international ethics. • Emotion regulation in global teams. • Emotion and international communication.	Van de Laar and De Neubourg, 2006; Jeffery, 2011; Graham, 2014; Hinds et al., 2014

Emotion can be a temporary state or a stable disposition. The difference is that dispositional emotion is cultivated in the early stage of life by an individual. It is stable in regard to its impact across the life course, such as describing one’s personality or trait, not vary with the environment change (Helson and Klohnen, 1998). The state of emotion is of its experienced nature (Russell and Barrett, 1999) and implies how a person sees his or her relationship to the environment and how a person interprets his or her circumstances (Smith and Ellsworth, 1985, p. 831). *Appraisal theory* assumes that an individual has a tendency to perceive new events and objects that determine the emotion. Appraisal tendencies explain the emotion and its impacts on shaping a decision-maker’s judgement and the subsequent choice of behaviour (Lerner and Keltner, 2000). Lerner and Keltner (2000) research initially looks at dispositional emotion but their finding demonstrate that appraisal induces emotion, no matter it is a stable disposition or a temporary state.

Entrepreneurs make decisions relying both on “the head (cognition) and on the heart (emotion)” (Cardon et al., 2012). They should be equipped with a balanced thinking style, synergising linear thinking (analytic, rational, and logical) focussing on “how do people think?” and non-linear thinking (intuitive, creative, and emotional) focussing on “how do people feel?” to “screen” the situation that they are embedded in Groves et al. (2011). Nonetheless, different emotion varies in their impacts on the outcomes of entrepreneurial activities. Positive emotion influences opportunity evaluation positively but decreases opportunity exploitation negatively; whereas, negative emotions constrain opportunity evaluation and opportunity exploitation (Grichnik et al., 2010). To be specific, fear, anger, and joy are different emotions with different impacts on an individual’s opportunity evaluation and their sequential tendency to exploit the opportunity (Welpe et al., 2012). Fear differs from other emotions, such as anger, happiness, and hope, in particular, influencing opportunity evaluation decisions though impacting on risk perceptions and risk preferences (Foo, 2011). As such, entrepreneurs with negative emotions are less likely to judge the new venture as an opportunity (Li, 2011).

By comparison, only a few IB scholars initiate the conversation on emotion and internationalisation: Meyer and Gelbuda (2006) and Van de Laar and De Neubourg (2006) argue that emotions moderate various aspects of the IP of the firm. Van de Laar and De Neubourg (2006) made the first attempt to explain emotions and their influence on Foreign Direct Investment (FDI) decisions. Their empirical research illustrates emotions as a function of personal factors, such as relations, involvement, interest, experience, and language competencies. As a result, managers’ emotional attachment to a project, a business relationship, or a country is relevant to investment decisions. Inspired by this study, Meyer and Gelbuda (2006) connect emotion to the IP model (1977). Their conceptualisation is to integrate emotion and investment decisions. The individual decision-maker’s emotions have an impact on influencing the firm’s investment and future commitments. Following the arguments building upon the premise that emotion and the IP intersect, this study questions their findings in treating emotions in general, which result in the drawback of failing to make distinctions between an emotional disposition and emotional state and positive emotions and negative emotions. In particular, the measurement of emotion is not convincing given the fact of treating emotion in general. It leads to their empirical finding being limited to its theoretical development.

This study proposes that negative emotion weights importantly in the context of making internationalisation decisions. The reason is that an individual decision-maker may feel unpleasant when estimating uncertainties (Isen et al., 1982). An individual is more likely to experience negative emotions strikingly in stressful situations (Gray, 1987). Engagement with an unfamiliar environment, such as the new international market, whereby one struggles to understand or predict interaction causes one to experience a cognitive state of uncertainty along with related anxieties that influence one other (Gudykunst, 1998). It can undermine the effectiveness of individual behaviour significantly. Uncertainty is involved in making the primary judgement, such as evaluating the evidence or experiencing the feelings associated with the judgement process. In addition, people respond to risk at two levels: cognitively, evaluating the threat posed by that risk is experienced during the decision-making process, and emotionally (Hastie, 2001, p. 670). Additionally, a focus on a stable dispositional emotion may not be appropriate in understanding how individuals perceive the internationalisation environment. The reason is because of the stable impact of the dispositional emotion, which is not varying with moving to a new environment, for example, the internationalisation environment. By comparison with a dispositional emotion, the entrepreneur’s emotions are event-generated and relevant to the object in the context concerning evaluation (Foo, 2011).

## Fear of Failure and Making Internationalisation Decisions

It is meaningful to look at fear, among other emotions, because the international market involves more risk, uncertainty, and complexity compared to a domestic market (Liesch et al., 2011). It should be carefully noted that fear can be a stable disposition or an emotional state. Fear is described as an unpleasant state that demands extreme amounts of effort (Smith and Ellsworth, 1985, p. 834). Appraisals of uncertainty and situational control induce fear. For instance, an individual fears when he/she senses the need for safety is not met, and such factors are beyond one’s control (Smith and Ellsworth, 1985). Fear means an emotional reaction to a threat (Gray, 1987), which influences decision-making, cognitive activity, behavioural responses (Damasio, 1994), and well-being (De Castella et al., 2013). Fearful people are more likely to perceive greater risk when moving into new environments (Lerner and Keltner, 2000). They demonstrate negative risk estimates and risk-averse choice preference for risk-free options over uncertain ones. Indeed, fear is the emotion linked to the appraisal of risk, uncertainty, and control, and this is equally true for both a dispositional emotion and a state emotion. Furthermore, uncertainty is a known factor that underpins fear. Uncertainty is derived from making the primary judgement, i.e., evaluating the evidence or experiencing the feelings associated with the judgement process (Hastie, 2001, p. 670). In the entrepreneurship literature, fear is a significant emotional factor in the entrepreneurial process (Baron, 2008), reflected in an individual’s evaluation of the riskiness of a specific opportunity (Foo, 2011), and further reduced the positive impact of opportunity evaluation (Welpe et al., 2012).

Instead of looking at fear generally, this study chooses a specific fear, fear of failure, which is likely to be triggered by the IP itself. First, fear of failure is important in the context of entrepreneurship and internationalisation because both can be seen as an achievement context for an individual. An achievement context/situation means “a situation in which an individual is responsible for an uncertain outcome, and this outcome will be evaluated at an excellence standard” (Atkinson, 1957, p. 360). In this situation, an individual’s need for achievement is strongly related to their choice of risk activities and the capacity to bear uncertainty and satisfy the incentives they seek by taking risks. Motivation drives an individual’s risk-taking behaviour, explaining why people choose tasks of different levels of difficulty (McClelland and Watson, 1973). Indeed, not every individual perceives starting up a business or an internationalisation process as a “must.” Some may be satisfied with other safe career choices or just running the business in a domestic market, which contains more familiarity when compared to the international market. Therefore, an individual’s motivation is key to explaining the fear of failure and one’s subsequent choice of a task in the achievement context, such as entrepreneurship or internationalisation. An individual’s motivation is an important factor in encouraging entrepreneurs to start international activities and in subsequently choosing the scale and scope of operations by firms in international markets (Zahra et al., 2005), where the internationalisation context requires decision-makers’ risk-taking behaviours (Clarke and Liesch, 2017).

Hope of success and fear of failure, as two motivating factors, are essential in the achievement context, linking to one’s risk-taking behaviours (Decharms and Dave, 1965). They suggest that the subject’s expectations concern their skills. On the one side, an individual’s avoidance motivation is associated with fear of failure, which undermines his/her engagement in the task (Elliot and Harackiewicz, 1996). For example, some individuals are more likely to be cautious safe and prefer achievable tasks in order to avoid potential failures rather than choose difficult tasks. Individuals with strong achievement motivation will have a more satisfying performance and ultimately maximise success and minimise failure (Decharms and Dave, 1965). By contrast, achievement motivation links to emotion (Weiner, 1985). It implies that an individual needs to feel pride in themselves to pursue and achieve something meaningful for themselves (Conroy et al., 2007). In turn, fear of failure shapes how an individual perceives achievement situations: they may be motivated to take actions to work hard to achieve the task or the opposite (Heckhausen and Heckhausen, 1991).

A concern is raised here: an individual may experience fear unconsciously and may not be aware of what he/she fears (Loewenstein et al., 2001). Differently, fear of failure may pinpoint the “failure,” as an object, guiding its impact. The internationalisation process implies operating in a continuously changing environment, going from domestic conditions to international markets, whilst possibly clinging to the existing organisational routines, and realising the company’s “unfitness” at the start of internationalisation (Sapienza et al., 2006). New market entry failure means the venture’s decreased involvement because the economic threshold set by the decision-maker/entrepreneur is not met (Ucbasaran et al., 2013). Concerning the internationalisation of a firm, failure is defined as “the venture’s unexpected decreasing involvement in international activities” (Nummela et al., 2016, p. 53) that include numerous de-internationalisation phenomena, such as divestment and export withdrawal. Whilst in some cases, this could be a strategic choice to optimise the firm’s operations; in other cases, such outcome signals lowered international commitment (Tra̧pczyński and Tra̧pczyński, 2016), and have a detrimental effect on the overall firm performance (Sapouna et al., 2018). The author suggests that decision-makers may perceive the “failure” object differently in different circumstances, depending on their own self-value, and the corresponding strategic choice for their firm’s internationalisation activities. Furthermore, fear of failure has different meanings to an individual decision-maker. It can be an important motivating factor that determines one’s choice about entrepreneurship or impedes entrepreneurship activities. Some scholars hold the view that fear of failure itself is a motive for individuals to avoid disappointment and the emotion of shame and embarrassment (Carsrud and Brännback, 2011). Fear of failure may stimulate an entrepreneur to put more effort into achieving their goals (Hayton et al., 2013), in particular, when an individual has high standards for success (Morgan and Sisak, 2016). Fear of failure is significant to entrepreneurs among other fearful emotions (Cacciotti and Hayton, 2015).

This research is aimed to contribute to the ongoing discussion on the fear of failure in line with other internationalisation studies. Fear of failure has received some attention in the IB literature. Alon et al. (2013) investigated the internationalisation of Chinese entrepreneurial firms and found that fear of failure decreases an entrepreneur’s likelihood to export. In the other study, Lafuente et al. (2015) explored the phenomenon from export entry to de-internationalisation through the lens of entrepreneurial attributes. They found that the entrepreneur’s fear of failure correlated with de-internationalisation, but not with export entry or export sustainability. Here, their finding is quite contradicted with Alon et al. (2013). Moreover, this study questions both studies’ research measurement of fear of failure as a stable disposition. Little attention has been given to exploring how an individual decision-maker experiences this emotion and how it impacts the IP of a firm.

## Implication for Future Research

The current dynamic business environment is encompassed with a higher level of complexities. As the author of the IP model states, “we cannot become psychologists ourselves, but we can apply psychological findings on our research issues and take a closer look at the micro-foundations of global strategy” (Vahlne, 2020, p. 246). Motivated by the research call, this manuscript thereby proposes that the concept of fear of failure can be applied to the IP model (2017), which is shown on the conceptual framework (Figure 2). This manuscript is limited to offering conceptual insights only, and so it encourages future research to empirically investigate the fear of failure in the IP by capturing the voice of decision-makers/business owners/entrepreneurs who could share their personal internationalisation experience. This conceptual manuscript, while serving as a starting point of the elaboration on the fear of failure in IB, offers directions for future research.

First, internationalisation decisions made by individual decision-maker to expand their ventures across borders are a fruitful context for in-depth exploration of fear of failure. Decisions made in an unfamiliar and challenging business environment are filled with risk and uncertainty (Liesch et al., 2011). The decision-maker who is potentially overwhelmed by unwanted externalities faces complex challenges in a constantly changing landscape. Failure in an overseas market disappoints the expectations of the decision-maker. Firms can also exhibit aversion to the obstacles of internationalisation, due to fear of future competition, insufficient resources, and perceiving the cost of establishing international networks with foreign partners as high (Westhead, 2008). This study believes that the internationalisation environment is sufficiently challenging and dynamics that it may trigger cognitive and emotional experiences of fear of failure and ultimately affect making internationalisation related decisions. Despite this evidence of the effects of fear of failure on the exporting behaviour, fear of failure may also lead to social stigma in the context of internationalisation activities (Alon et al., 2013), although little is known about how fear of failure impacts on making internationalisation decisions.

Second, the conceptualisation of fear of failure is likely to shed light on what is happening at the early stages of the internationalisation of a company. The effect of the fear of failure could subside once business owners gain more knowledge, experience and are able to strengthen their networks in international markets. This is worthy of further investigation. It would be promising to explore research questions that arise: how decision-makers experience the emotional and cognitive aspects of fear of failure when making internationalisation decisions for a firm? It is worthy looking at how they think and how they feel about this situation while making the decision. Moreover, the next step could explore the behavioural response to the fear of failure and ultimately trigger a change. It may uncover effective strategies to deal with the fear of failure.

Third, fear of failure is a context-sensitive phenomenon (Cacciotti et al., 2016). Decision-makers from emerging economies could offer a fruitful context for investigation due to their limited international networks (Ellis, 2011) and socio-cultural pressures in the domestic environment (Alon et al., 2013). The social context and institutional environment of China could be a productive environment in which to proceed with an empirical exploration of fear of failure. China has a long history of economic isolation before Chairman Deng Xiaoping implemented the “China’s policy of opening up to foreign business” (also called “open-door” policy) in 1978 (Wei, 1995), geopolitical risk uncertainty in China (Wang et al., 2021) and it lacks an export culture (Alon et al., 2013). Although China currently is a major player internationally, its success is attributed to state-owned enterprises’ FDI (Tang, 2019) while indigenous firms seem to lag behind due to poor decisions and “rushing in” to internationalise (Naudé, 2009). Fear of failure is also a dominant phenomenon in Chinese societies due to the notion of “face” highlighted in eastern cultures (Zane and Yeh, 2002). The fear of losing face is an important aspect of decision-making (Murray, 1999). Fear of losing face in Chinese societies can guide us to explore fear of failure underpinning its social aspect as applied to a commercial environment. Hence, future research could look at the social context with weak support to lead the empirical exploration of this phenomenon.

Fourth, future research is encouraged to research the fear of failure and the IP model by considering the COVID-19 phenomenon. COVID-19 has been last for 2 years which is associated with businesses risks (Yue et al., 2020; Li et al., 2021) and mental health symptoms, i.e., a high prevalence of anxiety, depression, and insomnia (Chen et al., 2021; Pappa et al., 2021; Zhang et al., 2022). Although COVID-19 has reached researchers’ attention on IB policy (Van Assche and Lundan, 2020) and strategy (Verbeke and Yuan, 2021), there is no IB empirical research that explored the fear of failure. International human resource management literature has noticed two key important issues accordingly: collaborating under stress, health, and safety (Caligiuri et al., 2020). Therefore, this study calls upon future research to consider the decision-maker’s wellbeing and fear of failure in the internationalisation in light of the COVID 19 situation.

Regarding the methodological foci, a qualitative investigation is a priority consideration to study this topic that includes the interview method and interpretative phenomenological analysis (Dong, 2019). Additionally, future research could explore fear of failure by collecting decision-makers/business owners/entrepreneurs’ Twitter messages and applying machine-learning techniques, such as text mining and AI sentiment (Song et al., 2022) and content analysis of web information (Li, 2013).

## Conclusion

This manuscript adds richness to the internationalisation literature to integrate the emotional perspective. In addition, it integrates a new concept, fear of failure, to reignite the discussion of “connecting people to the internationalization” and “emotion and the process of internationalisation.” The understanding of the role of the individual-level decision-maker in their firm’s internationalisation process is strengthened by leveraging off the entrepreneurship literature by considering a new individual-level factor, fear of failure. Furthermore, it reviewed various bodies of literature on emotion, specifically, fear and fear of failure, and their application to the entrepreneurship literature. This manuscript contributes to clarifying the nature of an emotional state and a dispositional emotion. It supports the “experience” view of fear of failure (Hayton et al., 2013; Cacciotti et al., 2016) to capture emotion, cognition, and action in the entrepreneurship process and suggests that the experience view of fear of failure is feasible as a lens through which to explore internationalisation. Fear of failure is a crucial emotional experience linked to an individual’s perception of risk and uncertainty and their choice of action. To understand the decision-maker’s choice of engaging in the internationalisation of their firm, there is a need to uncover the fear of failure experience.

This manuscript has implications for practitioners. Generally, individuals tend to see the fear of failure as an emotion that makes negative impacts. We highlight that fear reactions may be an unconscious experience when individuals are not aware of what they fear (Loewenstein et al., 2001). It is of benefit to decision-makers to understand why they experience fear of failure in deciding internationalisation and the diverse way in which fear of failure may be manifested in this scenario. This is especially applicable to the situation when entrepreneurs may succumb to fear of failure, for example, opting to exit export markets. We suggest building awareness about the fear of failure. Generally, individuals are reluctant to talk about their difficult emotions or emotional hardship, such as fear of failure. However, we cannot deny that consequences of the fear of failure include emotional impacts of stress, anxiety, worry, and cognitive disruption, and this has impacts on decision-making. Awareness of the fear of failure and understanding its mechanism is the road to finding ways to manage it instead of going through this experience silently.

## Author Contributions

RKD contributed to conception and design of the study, wrote the draft of the manuscript, contributed to manuscript revision, read, and approved the submitted version.

## Conflict of Interest

The author declares that the research was conducted in the absence of any commercial or financial relationships that could be construed as a potential conflict of interest.

## Publisher’s Note

All claims expressed in this article are solely those of the authors and do not necessarily represent those of their affiliated organizations, or those of the publisher, the editors and the reviewers. Any product that may be evaluated in this article, or claim that may be made by its manufacturer, is not guaranteed or endorsed by the publisher.
